# The Probable Infectious Origin of Multiple Sclerosis

**DOI:** 10.3390/neurosci4030019

**Published:** 2023-09-07

**Authors:** Remi L. Landry, Monica E. Embers

**Affiliations:** 1 Department of Tropical Medicine, Tulane University School of Public Health and Tropical Medicine, New Orleans, LA 70112, USA; rlandry10@tulane.edu; 2 Division of Immunology, Tulane National Primate Research Center, Tulane University Health Sciences, Covington, LA 70433, USA

**Keywords:** multiple sclerosis, Epstein–Barr virus (EBV), human herpesvirus 6, varicella-zoster virus, cytomegalovirus, *Helicobacter pylori*, *Chlamydia pneumoniae*, *Borrelia burgdorferi*, neuroinflammation

## Abstract

Multiple sclerosis (MS) is an immune inflammatory disease that causes demyelination of the white matter of the central nervous system. It is generally accepted that the etiology of MS is multifactorial and believed to be a complex interplay between genetic susceptibility, environmental factors, and infectious agents. While the exact cause of MS is still unknown, increasing evidence suggests that disease development is the result of interactions between genetically susceptible individuals and the environment that lead to immune dysregulation and CNS inflammation. Genetic factors are not sufficient on their own to cause MS, and environmental factors such as viral infections, smoking, and vitamin D deficiency also play important roles in disease development. Several pathogens have been implicated in the etiology of MS, including Epstein–Barr virus, human herpesvirus 6, varicella-zoster virus, cytomegalovirus, *Helicobacter pylori*, *Chlamydia pneumoniae*, and *Borrelia burgdorferi.* Although vastly different, viruses and bacteria can manipulate host gene expression, causing immune dysregulation, myelin destruction, and neuroinflammation. This review emphasizes the pathogenic triggers that should be considered in MS progression.

## 1. Introduction

Among chronic neurodegenerative diseases of the central nervous system (CNS), multiple sclerosis (MS) is one of the most prevalent. Affecting approximately 2.8 million individuals worldwide, multiple sclerosis is a chronic demyelinating autoimmune disease, the cause of which is still unknown [1]. The incidence and prevalence of MS varies significantly between different populations and geographic regions. MS is most common in people of Northern European ancestry, with an estimated prevalence of approximately 100–150 cases per 100,000 people in this population [1]. In contrast, MS is much less common in people of African, Asian, and Hispanic ancestry, with estimated prevalence rates of 8–10 cases per 100,000 people in these populations [1]. Globally, MS has a female preponderance, with women being twice as likely to have MS. In some countries, the ratio of women to men with MS is as high as 4:1 [1]. The reasons for these differences in MS prevalence are not yet fully understood, but it is believed that both genetic and environmental factors play roles. 

The onset and progression of MS varies from person to person, meaning that it is a heterogeneous disease requiring synthesis of contributing factors. The indications of MS encompass impaired motor function, fatigue, eye movement disorders, cognitive impairment, ataxia, and dementia. The clinical progression of MS is somewhat unpredictable, with distinct clinical phases based on the pattern of disease activity and accumulation of disability over time: relapsing-remitting MS (RRMS), primary progressive MS (PPMS), and secondary progressive MS (SPMS) [2]. RRMS is the most common form of the disease and is characterized by acute relapses or exacerbation of symptoms, followed by periods of partial or complete recovery [2]. The frequency and severity of relapses can vary widely between individuals and may be influenced by a range of factors, including genetics, environmental factors, and comorbidities. SPMS is a subtype of the disease that typically develops in people with RRMS after a period of several years. SPMS is characterized by gradual accumulation of disability and progressive worsening of symptoms, with or without superimposed relapses [2]. PPMS is a less common subtype of the disease, accounting for approximately 10–15% of all cases [3]. PPMS is characterized by steady progression of disability from onset of disease, without relapses or periods of remission.

The clinical progression of MS is typically monitored using a range of tools and measures, including neurological exams, imaging studies, and patient-reported outcome measures, such as the Expanded Disability Status Scale or Multiple Sclerosis Functional Composite [4]. MRI can be used to detect areas of inflammation and demyelination, which are characteristic of MS lesions [5]. The location and extent of these lesions can vary depending on the subtype of MS and stage of the disease.

Another important diagnostic tool is a neurological exam, which can include tests to assess the strength, reflexes, coordination, and sensation in different parts of the body. In addition to these tests, a lumbar puncture may be performed to obtain a sample of cerebrospinal fluid (CSF). The analysis of CSF can help to identify abnormalities that are characteristic of MS, such as the presence of oligoclonal bands, which are indicative of an abnormal immune response [6]. Blood tests may also be performed to rule out other conditions that can cause similar symptoms to MS, such as infections, vitamin deficiencies, and autoimmune disorders. A diagnosis of MS is typically based on a combination of clinical evaluation, medical history, and the results of diagnostic tests. These measures can help to track changes in disease activity and disability over time and guide treatment decisions. While there is currently no cure for MS, a range of disease-modifying therapies are available that can help to slow disease progression and reduce the frequency and severity of relapses. Treatment decisions are typically guided by disease subtype and activity and patient-specific factors such as comorbidities and lifestyle factors.

## 2. Pathogenesis

One of the key pathological features of MS is the presence of inflammatory lesions or plaques in the white matter of the CNS. These plaques are characterized by the infiltration of immune cells, such as T cells, B cells, and macrophages, into the CNS [7]. These cells release cytokines and other inflammatory mediators that activate resident glial cells, such as astrocytes and microglia, leading to a cascade of events that ultimately result in demyelination and axonal damage. The demyelination of axons in MS is thought to result from a combination of direct immune-mediated attack on myelin and the indirect effects of inflammation on oligodendrocytes, the cells responsible for producing myelin in the CNS. Demyelination leads to the exposure of axons, which can then become damaged due to a range of mechanisms, including oxidative stress, energy failure, and neurodegeneration that leads to brain atrophy [8]. The chronic inflammation associated with MS can also lead to the accumulation of gliosis in the CNS [9]. Gliosis is characterized by the proliferation of astrocytes and microglia, along with the deposition of extracellular matrix proteins such as collagen and fibronectin. The accumulation of tissue damage can disrupt the normal architecture of the CNS, and also impede remyelination and axonal regeneration. Plaques in the white matter are characterized by active destruction of axons and their myelin sheaths, which ultimately results in the development of irreversible neurological symptoms in the affected individual [7]. In addition to white matter lesions, MS can also affect the gray matter, particularly in later stages of disease. Gray matter atrophy is associated with cognitive impairment and disability in MS, and it is thought to result from a combination of axonal damage, neuronal loss, and gliosis [10].

### 2.1. Nature vs. Nurture

It is generally accepted that the etiology of MS is multifactorial and believed to be a complex interplay between genetic susceptibility, environmental factors such as smoking and obesity, and infectious agents that lead to immune dysregulation and CNS inflammation. It may be extremely valuable to define disease phenotypes based on the underlying pathologic mechanisms to achieve success with the personalized approach. Defining disease subtypes based on biology rather than on clinical manifestations should improve the validity of clinical trials, as drugs target the mechanism of disease and not the clinical stage. A recent study used machine learning to classify MS based on pathological features rather than disease subtype (RRMS, SPMS, and PPMS) for magnetic resonance imaging (MRI) [11]. The researchers used a training dataset of 6322 MS patients with every clinical subtype to define MRI-based subtypes and an independent cohort of 3068 patients for validation. Based on the earliest abnormalities, MS subtypes were defined as cortex-led, normal-appearing white matter-led, and lesion-led. People with the lesion-led subtype had the highest risk of confirmed disability progression and relapse rate [11]. People with the lesion-led MS subtype showed positive treatment responses in selected clinical trials [11]. These findings could provide important clues regarding etiology, either genetic or microbial.

While the exact cause of MS is not yet fully understood, it is believed that risk of developing the disease is strongly influenced by an individual’s genetic makeup. For decades, the only genes found to be risk factors were the human leukocyte antigen (HLA) genes. These genes strongly affect the immune reactivity of T cells. Certain variations of the HLA gene, such as HLA-DR2 and HLA-DQw1, have been linked to increased risk of developing MS, particularly in people of European descent [12,13]. HLA molecules play a pivotal role in presenting antigens to T cells. Certain haplotypes have been associated with increased susceptibility to disease, suggesting their involvement in initiating or perpetuating the autoimmune response against CNS components. Molecular mimicry adds a layer of intricacy to the HLA narrative [14]. Pathogens, which bear structural resemblances to self-antigens, can trigger cross-reactive immune responses. When considering HLA haplotypes, specific combinations might have a greater propensity to present both pathogen-derived and self-antigens, enhancing the likelihood of cross-reactivity [15,16]. This scenario could potentially fuel the characteristic autoimmune cascade of MS, as the immune system encounters both foreign and self-antigens through shared HLA context. The implications of this interplay are profound. Certain HLA haplotypes might act as conduits for molecular mimicry-driven cross-reactivity, inadvertently priming the immune system to target self-components in the CNS due to structural resemblances to pathogenic antigens [17]. This phenomenon could substantially influence disease susceptibility and progression, amplifying the autoimmune response against CNS tissues.

More recently, genome-wide association studies (GWAS) identified more than 200 genetic variants associated with increased MS risk, with approximately 30 associated with the major histocompatibility complex (MHC) locus [18]. Many of these variants are located in or near genes that are involved in immune function and regulation, such as the MHC region on chromosome 6, which contains several genes involved in antigen presentation and T-cell activation. Other genetic variants that have been identified via GWAS have well-ascribed functions in other aspects of immune regulation, such as cytokine signaling and T-cell differentiation, but some are associated with mitochondrial function or myelin structure. For example, variants in the interleukin 7 receptor (IL7R) gene have been shown to increase the risk of developing MS, likely by affecting T-cell homeostasis and activation [19,20,21]. Large-scale whole-genome sequencing studies have also identified rare genetic variants that are associated with increased risk of developing MS [22]. These variants are less common in the general population, but many are located in or near genes that are involved in immune function and regulation, similar to the common variants. Despite the progress made in identifying genetic variants associated with MS, the majority of the heritability of the disease remains unexplained. This suggests that there are likely many more genetic variants that contribute to MS risk, and that the genetic architecture of the disease is likely to be very complex.

**Figure 1:**
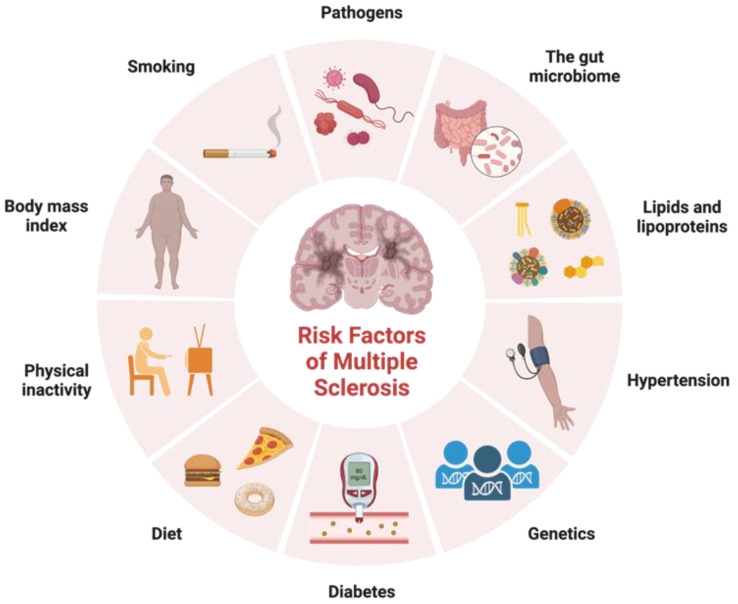
Risk factors for multiple sclerosis. Adapted from “Risk Factors of Dementia”, by BioRender.com. Accessed on 17 February 2023. Retrieved from https://app.biorender.com/biorender-templates.

In addition to identifying variants in immune function that contribute to MS risk, genetic studies have also provided insight into the biology of the disease. These genetic factors are not sufficient on their own to cause MS, and environmental factors such as viral infections, smoking, and vitamin D deficiency also play important roles in disease development (Figure 1). Factors that exhibit correlations with certain ethnic groups or MS subtypes have emerged as intriguing associations that reflect the intricate interplay between genetic predisposition, environmental influences, and disease susceptibility. Variants in genes involved in vitamin D metabolism and regulation have been associated with increased risk of developing MS, suggesting that vitamin D deficiency may play a role in the disease. Studies have shown that low levels of vitamin D are associated with increased risk of developing MS and that vitamin D supplementation may help to reduce disease activity and disability in people with MS [23]. Noteworthy observations point to a higher prevalence of MS among individuals of Northern European descent, attributed in part to paler skin facilitating efficient vitamin D synthesis upon sunlight exposure [23]. Smoking is another environmental factor that has been linked to increased risk of developing MS. Studies have shown that people who smoke have a higher risk of developing MS, and that smoking may also contribute to more severe disease progression [24]. Diet has also been proposed as a potential environmental factor in MS etiology. The intriguing prospect of the gut microbiota impacting disease subtype underscores its potential to modulate MS progression differentially across patient groups. Cultural dietary practices often exhibit ethnic nuances, which extend to their influence on the gut microbiota composition. A diet high in saturated fat and low in fruits and vegetables has been associated with increased risk of developing MS, while a diet rich in omega-3 fatty acids and antioxidants may help to reduce disease activity and disability [25]. As research advances, a refined understanding of these complex relationships holds the potential to unveil novel insights into the mechanisms underlying MS etiology, ethnic disparities, and disease subtypes.

### 2.2. Microbes and Infectious Agents

Among the environmental factors that have been implicated in the development of MS are infectious agents and microbes. The gut microbiome, which refers to the community of microorganisms that inhabit the human gastrointestinal tract, has been increasingly recognized as an important player in various physiological and pathological processes, including immune regulation, metabolism, and neurological function. The gut microbiome is known to interact with the host immune system through gut-associated lymphoid tissue (GALT), which is the largest immune organ in the body and implicated in the pathogenesis of various autoimmune and inflammatory diseases, including MS [26]. Recent studies have shown that the gut microbiome in MS patients differs from that in healthy individuals, with alterations in the abundance and diversity of certain bacterial species. For instance, MS patients have been shown to have decreased abundances of some beneficial bacteria, such as *Akkermansia muciniphila*, *Faecalibacterium prausnitzii*, and *Butyricimonas* spp., and increased abundances of some pro-inflammatory bacteria, such as *Eubacterium hallii*, *Ruminococcus gnavus*, and *Parabacteroides distasonis* [27,28]. These alterations in the gut microbiome have been proposed to contribute to the development and progression of MS through several mechanisms, including modulation of the immune response, alteration of blood–brain barrier (BBB) integrity, and production of neuroactive metabolites [29]. The gut microbiota and its prominent metabolic products, known as short-chain fatty acids (SCFAs), stand as pivotal entities in maintaining gut homeostasis and have implications in metabolic disease occurrence. SCFAs, such as acetate, butyrate, and propionate, are formed by gut microbial fermentation of dietary fibers and carbohydrates [30]. These SCFAs play an immunomodulatory role, with potential to affect the CNS. These substances can play roles in regulating blood pressure, GI function, and immune system function [31] Interestingly, decreased SCFA levels have been reported in patients with diseases such as Parkinson’s, Alzheimer’s, and anorexia nervosa [32]. In individuals afflicted by MS, there also appears to be an impact on the enteric nervous system (ENS). The ENS occupies a central role in orchestrating the intricate interplay between the gut microbiota and gastrointestinal homeostasis. The ENS plays an essential role in maintaining gut barrier integrity and immune surveillance [33]. SCFAs can influence ENS function. Notably, the ENS, constituting a substantial portion of the peripheral nervous system, remarkably mirrors the components and functionalities of the CNS. Signs of ENS disorder have been found in various neurodegenerative conditions, such as amyotrophic lateral sclerosis (ALS), Alzheimer’s, Parkinson’s, and MS [34]. Given the potential role of the gut microbiome in MS, there has been growing interest in developing microbiome-based therapies for this disease. These approaches include the use of probiotics, prebiotics, fecal microbiota transplantation, and dietary interventions aimed at modulating the gut microbiome. However, further research is needed to better understand the complex interactions between the gut microbiome and MS and to identify the most effective and safe microbiome-based interventions for this disease. Mucosal-associated invariant T (MAIT) cells also play a role in the gut–brain axis and the development of autoimmune neuropathology [35]. A recent study demonstrated that MAIT cells were significantly more activated in people with MS compared to healthy donors in response to yeast strains isolated from fecal samples. In addition, immunofluorescent staining of post-mortem brain tissues from individuals with the secondary progressive form of MS showed that MAIT cells cross the BBB and produce pro-inflammatory cytokines in the brain [36]. The process appears to begin with dysbiosis in the gut, followed by microbe-induced activation of MAIT cells in a TCR-dependent or independent manner. These cells become activated by IL-23 released from dendritic cells and monocytes. The activated MAIT cells then migrate to the CNS, cross the BBB, and release pro-inflammatory cytokines such as IL-17, GM-CSF, and IFN-ɣ, leading to neuronal damage [37]. MAIT cells also secrete CCL20, which recruits other CCR6-expressing T cells across the BBB, exacerbating neuroinflammation [38].

In addition to the gut microbiome, several studies have implicated microbial infections as determinants of MS risk. An ongoing debate persists as to whether microbial infections trigger MS in genetically predisposed individuals. Epigenetic mechanisms in the context of MS constitute a captivating terrain of research, and their intricate interplay with microbial involvement offers a multifaceted perspective on the disease’s etiopathogenesis [39]. Epigenetics involves modifications to DNA and histone proteins that orchestrate gene expression patterns without altering the genetic sequence. Epigenetic mechanisms assume a pivotal role in mediating the interplay between genetic susceptibility and environmental influences. Microbial involvement has been shown to modulate epigenetic markers [40]. DNA methylation, a prominent epigenetic modification, entails the addition of methyl groups to DNA strands, often resulting in gene silencing [41]. In the context of microbial exposure, DNA methylation patterns can be dynamically altered, impacting genes associated with immune responses and inflammation [42]. Microbial interactions might provoke epigenetic changes that skew the immune balance, potentially contributing to the immune dysregulation that characterizes MS. Histone modifications offer another layer of epigenetic orchestration. Microbial involvement has been shown to modulate epigenetic markers [40]. This modulation might significantly impact the equilibrium between regulatory and pro-inflammatory responses relevant to MS pathogenesis. Non-coding RNAs, encompassing microRNAs (miRNAs) and long non-coding RNAs (lncRNAs), stand as another dimension of epigenetic modulation influenced by microbes [43]. These molecules, although they do not encode proteins themselves, profoundly impact gene expression by binding to messenger RNAs, either inhibiting or facilitating their translation. Perturbed miRNAs and lncRNAs, sculpted in response to microbial cues, might markedly contribute to the immune dysregulation that characterizes MS.

The role of infectious agents is also supported by the “hygiene hypothesis,” which proposes that reduced exposure to pathogens early in life can increase the risk for autoimmune diseases like MS [44]. This is due to an imbalance in Th1 and Th2 immune responses. However, helminths and protozoans can act with different immunological mechanisms. Protozoans induce a strong Th1 response through the production of pro-inflammatory mediators such as IL-12, nitric oxide, and IFN-γ [45]. Helminths, on the other hand, induce a Th2 response characterized by the release of IL-4, IL-5, and IL-10 [46]. The infectious origin of MS considers whether infections induce or accelerate autoimmune diseases like MS. Numerous infectious agents have been suggested to play roles in the pathogenesis of MS, including viruses, bacteria, and parasites. Among the most widely studied are Epstein–Barr virus (EBV), human herpesvirus 6 (HHV-6), varicella-zoster virus (VZV), *Chlamydia pneumoniae*, and *Helicobacter pylori*; however, to date, no infectious agents have been proven to cause MS.

### 2.3. Treatments

Over the years, various treatments have been developed to manage MS, aiming to reduce relapses, slow disease progression, and alleviate symptoms. These treatments can be broadly categorized into disease-modifying therapies (DMTs) and symptomatic treatments [47,48]. Symptomatic treatments for MS aim to alleviate and manage specific symptoms, such as spasticity, pain, bladder dysfunction, and fatigue. These treatments include muscle relaxants, beta blockers, physical therapy, pain relievers, medications to improve bladder control, speech therapy, and anti-fatigue medications.

DMTs have revolutionized MS management by targeting aberrant immune responses. Their mechanisms of action vary, encompassing immunomodulation, immunosuppression, and immune reconstitution. Monoclonal antibodies (MABs) have gained significant attention for their precision in targeting immune cells and molecules involved in MS pathogenesis [49]. Interferon beta was one of the earliest DMTs approved for MS. Interferon beta is available in different formulations, including interferon beta-1a and interferon beta-1b, and works by modulating the immune response, reducing inflammation, and promoting a more balanced immune system [50]. It also helps to decrease the number and severity of relapses in RRMS. Ocrelizumab is another monoclonal antibody that targets B cells, a type of immune cell involved in the immune response that damages myelin. By depleting certain B cells, ocrelizumab has demonstrated efficacy in reducing relapse rates and delaying progression in RRMS and PPMS [49]. Other DMTs, like natalizumab and alemtuzumab, have shown promising results by modulating immune cell migration and function. Natalizumab was first approved in 2004 and is a monoclonal antibody that targets integrins on immune cells, specifically preventing immune cells from crossing the BBB and entering the CNS, and therefore reducing the inflammation seen in MS [51]. Alemtuzumab targets CD52, a protein found on the surface of various immune cells [49]. It leads to the depletion of lymphocytes, particularly T and B cells, followed by repopulation with new cells. This “resetting” of the immune system can help to reduce inflammation and the autoimmune response seen in MS. Glatiramer acetate, commonly known as Copaxone, is another DMT that is thought to work by altering the immune response [52]. It is believed to simulate myelin basic protein, a component of the myelin sheath that is targeted in MS. By doing so, it diverts the immune system’s attack away from myelin, helping to reduce inflammation and the frequency of relapses. Dimethyl fumarate is an oral DMT that exerts its effects through multiple mechanisms, including activation of the Nrf2 pathway, which is involved in cellular defense against oxidative stress [53,54]. This drug helps to reduce inflammation and oxidative damage in the CNS. Fingolimod was the first DMT approved for relapsing forms of MS [55]. It is an oral DMT that works by binding to sphingosine-1-phosphate receptors on immune cells, preventing them from leaving lymph nodes and entering the central nervous system [55]. This reduces the number of immune cells available to cause inflammation in the brain and spinal cord.

The aforementioned DMTs can have implications for how the immune system responds to infectious insult. Ocrelizumab, alemtuzumab, and fingolimod can suppress the immune system to varying degrees. However, the immunomodulatory effects of DMTs can inadvertently impact the host’s ability to respond to microorganisms, influence the reactivation of latent infections, and potentially increase susceptibility to various types of viral, bacterial, and fungal infections. Some DMTs, like natalizumab, can affect the immune system’s response to specific viral infections, particularly the John Cunningham virus (JC virus) [56]. This virus can lead to progressive multifocal leukoencephalopathy (PML), a rare but serious brain infection [56]. Other immunosuppressive DMTs may disturb immune surveillance, triggering reactivation of latent infections. Varicella-zoster virus and tuberculosis are notable examples of infections that can reactivate due to compromised immune control [57]. The complex interplay between MS DMTs, microorganisms, and latent infections underscores the need for a comprehensive understanding of both therapeutic benefits and potential immunological risks.

## 3. Viral Triggers

The search for a causal association between viruses and the development of MS spans nearly a century. In the late 19th century, Jean-Martin Charcot and Pierre Marie postulated an infectious etiology for MS [58]. Elevated titers of rubella and measles in the CSF of patients with MS were the first insights into the role of persistent infection in MS [59]. The viral hypothesis of MS was strengthened in the 1980s when an Italian study found higher titers of antibodies against Epstein–Barr virus (EBV) and herpes simplex 2 in patients with MS compared to healthy controls [60]. Other viruses have been implicated in the pathogenesis of MS, including varicella-zoster, herpes simplex 1 and 2, HHV-6, and cytomegalovirus (CMV). These viruses are thought to enter the brain and establish latent chronic infection. Different mechanisms have been hypothesized for the involvement of viruses in the pathogenesis of MS. The viruses involved are thought to trigger or reactivate an autoimmune response against the myelin sheath, leading to demyelination and neurodegeneration. It is also suggested that viruses can manipulate the gene expression of the host, leading to tissue damage and deregulation. The mechanisms are not mutually exclusive. Although the infectious origin of MS is a controversial topic, there is interest in the role of viruses as a potential risk factor in genetically susceptible individuals.

### 3.1. Epstein–Barr Virus

Epstein–Barr virus infection is one of the infections most consistently associated with MS. EBV is a ubiquitous, enveloped, double-stranded Herpesviridae virus that is transmitted via saliva. Similar to other herpes viruses, EBV infects and establishes lifelong infection in more than 95% of adults worldwide [61,62]. The virus enters the immune system via pharyngeal epithelial cells and infects B cells, where it may be propagated or enter latency for the life of the infected individual. In up to 75% of young adults and adolescents, primary infection presents as infectious mononucleosis [63]. EBV infection has been linked to several diseases that develop years after the primary infection. EBV infection may induce autoreactive T cells that cross-react with myelin antigens, leading to an autoimmune response and promoting the onset of diseases such as cancer, MS, and other autoimmune disorders [64].

Since the 1970s, there has been a growing persuasive body of epidemiological and immunological studies linking EBV to CNS disease development. EBV has evolved mechanisms to evade the immune system and counteract both host cell intracellular anti-viral processes and host immune responses [65]. Several mechanisms have been proposed to explain the link between EBV and MS, including molecular mimicry, wherein the immune system recognizes both EBV and myelin antigens, leading to an autoimmune response that damages myelin in the CNS. Another possible mechanism is that EBV may trigger an abnormal immune response in susceptible individuals, leading to the development of MS. In addition to these mechanistic links, studies have shown that EBV infection is associated with increased risk of relapse in people with MS and may also contribute to disease progression and disability [66].

In a systematic review of case–control studies, Ascherio and Munch found the incidence of MS to be significantly higher in EBV-seropositive individuals [67]. Recent meta-analyses also support a casual role of EBV infection and increased risk of developing MS. Researchers found that a higher proportion of MS patients had a history of infectious mononucleosis and seropositivity against EBV nuclear antigen and viral capsid antigen [68]. A comprehensive epidemiological study was recently published in which serial serum samples from US military personnel were analyzed. Researchers found a 32-fold increased risk of MS diagnosis in individuals who were EBV-seropositive compared to those who remained seronegative [66,69]. An associated risk for MS following infection was also found in cases of pediatric MS. Individuals who acquired EBV or infectious mononucleosis during adolescence or later were shown to be at 2–3-fold higher risk of developing MS compared to children infected early in life [69,70,71,72,73]. Conversely, a recent study from Stanford demonstrated that people with MS had significantly higher levels of antibodies to EBV compared to people without disease [74]. This study also found that people who developed MS at a younger age had higher levels of antibodies to EBV than those who developed MS later in life. Another study showed that individuals with MS had higher levels of antibodies to EBV than those without MS. The poliovirus receptor (PVR) risk SNP, which affects how the immune system responds, was associated with EBV DNA copy number and PVR [75]. The study suggested that EBV may trigger an abnormal immune response that leads to MS and that controlling EBV infection may help to prevent or treat the disease. In contrast to antibody-based studies, investigations using molecular methods like PCR on EBV DNA and RNA in blood, CSF, and saliva have shown only minor differences between controls and MS patients [76,77,78]. In situ hybridization and PCR studies on post-mortem brain tissue from MS patients have indicated the presence of EBV DNA in lesions, but the results are conflicting [79,80,81,82,83]. Taken together, EBV is a highly likely candidate for an etiologic agent in MS. However, much remains to be investigated regarding the mechanisms of pathogenesis [84].

### 3.2. HHV-6

Several studies have linked human herpesvirus 6 (HHV-6) with MS pathogenesis (Table 1). HHV-6 is a double-stranded virus that is known to establish lifelong latency in the human body after primary infection. Prior studies reported that more than 95% of adult populations in developed countries were seropositive for HHV-6 [85,86,87,88]. Two variants of HHV-6 have been recognized: HHV-6A and HHV-6B. HHV-6A has a preference for infecting neural cells and has been found in MS lesions [89,90]. HHV-6 has been implicated in a variety of neurological disorders, including encephalitis, chronic fatigue syndrome, mesial temporal lobe epilepsy, and MS [91]. HHV-6 has been suggested to trigger an autoimmune response in MS by inducing the expression of pro-inflammatory cytokines and activating autoreactive T cells. HHV-6 may also contribute to MS pathogenesis by promoting neuroinflammation, disrupting the blood–brain barrier (BBB), and causing direct damage to oligodendrocytes, the myelin-producing cells in the central nervous system. Some studies suggest that HHV-6 proteins may have cross-reactivity with myelin basic protein, which is a crucial component of the myelin sheath and could contribute to CD8+ T cell-mediated oligodendrocyte death [92].

HHV-6 was first regarded as a promising candidate for MS pathogenesis in the early 1990s. Sola et al. investigated antibody titers and found that MS patients had a significantly higher HHV-6-specific serum antibody titers than controls [93]. Epidemiological studies have suggested that HHV-6 infection may be more common in MS patients. Pormohammad et al. conducted a systematic review and meta-analysis focused on the association between HHV-6 and MS [94]. Out of the 39 included studies, 34 used molecular assays for surveying the relationship between MS and HHV-6. Compelling evidence includes a higher prevalence of viral DNA and proteins in MS plaques and CSF compared to healthy patients, indicating HHV-6 neurotropism [95,96]. The detection rates are higher in brain tissue samples than in serum or CSF. The meta-analysis concluded that HHV-6 increases the risk of MS; however, findings varied between studies using different sample materials and research methodologies for patients with MS and controls (Table 1). Only a few studies reported a significant difference between controls and MS patients. In addition, not much is known about the possible mechanisms by which HHV-6 might be involved in MS pathogenesis. A recent study reported that HHV-6 IgG is associated with increased risk of MS conversion and relapse [97]. These studies have provided some evidence that herpesvirus infection and host immunity are involved in MS disease course, but further research regarding the mechanisms is needed. Furthermore, the presence of viral RNA and proteins in periventricular lesions, which are commonly observed in MS, also supports the involvement of HHV-6 in MS pathogenesis [98,99]. Despite these findings, other studies have reported a lack of HHV-6 detection in MS.

**Table 1:** Characteristics of studies linking HHV-6 in MS pathogenesis.

Source	Year	Methods	Samples
Sola et al. [93]	1993	PCR, Southern blot, IFA	CSF, serum
Wilborn et al. [100]	1994	PCR, ELISA	Serum
Challoner et al. [99]	1995	Representational difference analysis	CNS tissue, PBL
Liedtke et al. [101]	1995	Nested PCR	CSF, serum
Sanders et al. [102]	1996	PCR	Tissue
Carrigan and Knox [103]	1997	--	--
Merelli et al. [95]	1997	PCR	PBMCs
Martin et al. [104]	1997	PCR, indirect immunofluorescent assay	CSF, serum
Soldan et al. [105]	1997	EIA, IFA	Serum
Ablashi et al. [106]	1998	PCR	CSF
Coates and Bell [107]	1998	PCR	Serum, CSF
Mayne et al. [108]	1998	PCR, nested PCR	Blood
Friedman et al. [109]	1999	PCR, immunohistochemistry	Tissue, CSF
Ongradi et al. [110]	1999	ELISA	CSF
Rotola et al. [111]	1999	Nested PCR	PBMCs
Ablashi et al. [112]	2000	PCR	CSF, sera, plasma, blood
Akhyani et al. [113]	2000	Nested PCR	PBL, serum, saliva, urine
Kim et al. [114]	2000	PCR	PBMC
Knox et al. [115]	2000	IFA, rapid HHV-6 culture assay	CNS tissue, blood
Alvarez-Lafuente et al. [116]	2002	qRT-PCR	Blood
Berti et al. [117]	2002	Nested PCR	Blood
Tejada-Simon et al. [118]	2002	PCR, nested PCR, Southern hybridization	CSF, blood
Xu et al. [119]	2002	ELISA	Serum
Al-Shammari et al. [120]	2003	Nested PCR, PCR	Serum
Cermelli et al. [121]	2003	Nested PCR	Tissue
Chapenko et al. [122]	2003	PCR, nested PCR	PBMCs, blood
Alvarez-Lafuente et al. [123]	2004	Quantitative RT-PCR	Blood, serum
Rotola et al. [124]	2004	Nested PCR	CSF, PBMCs
Derfuss et al. [125]	2005	ELISA	CSF/Serum, PBMCs
Fogdell-Hahn et al. [126]	2005	PCR	Blood, CSF
Höllsberg et al. [127]	2005	RT-PCR	Blood, saliva
Alvarez-Lafuente et al. [128]	2006	qRT-PCR	Serum
Alvarez-Lafuente et al. [129]	2007	RT-PCR	Serum, PBMCs
Virtanen et al. [130]	2007	Immunofluorescence avidity assays, multiplex PCR	Serum, CSF
Kuusisto et al. [131]	2008	PCR, ELISA	Serum, CSF
Alvarez-Lafuente et al. [132]	2009	qRT-PCR	PBMCs, serum
Mancuso et al. [133]	2010	RT-PCR	CSF
Behzad-Behbahani et al. [134]	2011	Nested PCR	Serum
Garcia-Montojo et al. [135]	2011	Quantitative RT-PCR	Blood, serum
Nora-Krukle et al. [136]	2011	Nested PCR, RT-PCR, ELISA	Plasma, serum
Virtanen et al. [137]	2011	Isoelectric focusing and immunofixation, affinity-driven immunoblot	Serum, CSF
Dominguez-Mozo et al. [138]	2012	qRT-PCR	Blood, serum
Ramroodi et al. [139]	2013	qRT-PCR	PMBCs, serum, saliva
Alenda et al. [140]	2014	SDS-PAGE, MALDI-TOF MS	CSF
Hon et al. [77]	2014	PCR	CSF, blood
Ortega-Madueño et al. [141]	2014	ELISA	Serum
Simpson et al. [142]	2014	qRT-PCR	Serum, CSF
Kofahi et al. [143]	2020	ELISA	Blood
Tao et al. [97]	2022	PCR, indirect immunofluorescence assay	Blood, serum

PCR = Polymerase chain reaction, IFA = Immunofluorescence assay, ELISA = Enzyme-linked immunosorbent assay, CSF = cerebrospinal fluid, PBL = Peripheral blood leukocytes, PBMCs = Peripheral blood mononuclear cells, EIA = Enzyme immunoassay, RT-PCR = Real-time PCR.

### 3.3. Varicella-Zoster Virus

Varicella-zoster virus is a highly contagious, ubiquitous alpha herpesvirus that causes two distinct clinical syndromes: varicella (chickenpox) and herpes zoster (shingles) [144]. VZV is transmitted through respiratory secretions or contact with skin lesions of an infected person. Characteristic of alphaherpesviruses, VZV establishes latency in the dorsal root or cranial nerve ganglia [145]. CNS complications of chickenpox are rare but can include myelitis, Reye’s syndrome, and meningitis [146]. VZV has been considered as a potential etiologic agent in MS, but studies have shown conflicting evidence for an association. MS patients have a higher incidence of history of VZV and increased antibody response to it compared to the general population [147,148,149]. Studies have shown detection of viral DNA in the CSF and brain tissue of patients with progressive disease; however, other studies have failed to confirm these findings [150,151]. Thus, VZV may play a role in the CNS inflammation seen in MS. Previous studies have indicated that reactivation of latent VZV might correspond with relapse in MS patients [152,153,154]. Sotelo et al. indicated a causal role for VZV in the pathogenesis of MS relapse [155]. Researchers found VZV in the CSF and blood of patients in relapse and noticed a decrease during disease remission. On the other hand, a systematic review found insufficient evidence to support the role of VZV infection in MS etiopathogenesis [156]. VZV is often detected during active phases of MS, although it is not clear if this finding is linked to the pathogenesis of MS or merely an incidental occurrence due to treatment or disease [154]. It has been proposed that VZV reactivation could trigger an autoimmune response in susceptible individuals, leading to demyelination and MS development or exacerbation. VZV reactivation is a recognized complication of the immunosuppressive therapies available for MS treatment, in particular with fingolimod [145]. In a similar fashion, immunosuppressive drugs used in MS therapy have been associated with viral encephalitis as a complication of herpes simplex virus types 1 and 2 (HSV-1 and HSV-2), two other alphaherpesviruses [157,158].

### 3.4. Cytomegalovirus

Cytomegalovirus, a ubiquitous herpesvirus, has garnered significant attention as a potential contributor to MS development and progression. CMV is a common virus that infects a large portion of the population worldwide, with some estimates suggesting between 50% and 80% of all adults in the U.S. are infected by the age of 40 [159]. However, the virus displays socioeconomic and racial disparities in the US population similar to those seen in EBV [160]. Like EBV, CMV is transmitted through saliva. Due to its prevalence and ability to establish lifelong latent infection, CMV has emerged as a viral candidate for MS pathogenesis. In two studies conducted on an Iranian group of MS patients, higher levels of CMV DNA were detected compared to the control group [161,162]. These results were supported by evidence showing CMV reactivation as a possible exacerbating factor in MS patients [163,164]. CMV infection elicits a robust immune response involving both innate and adaptive immunity. In individuals with MS, dysregulation of immune responses is a key feature of disease pathogenesis. Studies have suggested that the immune response triggered by CMV infection may contribute to the autoimmune processes underlying MS development. However, the precise mechanisms involved in CMV-induced immune dysregulation in MS necessitate further investigation. Many studies have reported a negative correlation between CMV seropositivity and MS diagnosis [165,166,167,168,169]. A comprehensive meta-analysis of 1341 MS patients and 2042 controls failed to establish a conclusive relationship between CMV infection and MS [170]. These variations in findings may be attributed to a phenomenon similar to that observed with the Epstein–Barr virus, where individuals who have never been infected with CMV have a lower risk of developing MS, while reactivation of latent CMV during the active MS phase could aggravate the existing damage.

## 4. Bacterial Triggers

Bacteria also play a potential role in the development of multiple sclerosis. It is possible that bacteria may trigger an abnormal immune response in susceptible individuals, leading to the development of autoimmune disorders such as MS. While the evidence linking bacterial infections to the development of MS is not conclusive, findings suggest that targeting the microbiome may be a potential avenue for developing new treatments for MS.

### 4.1. H. pylori

*Helicobacter pylori* is a common bacterial infection that has been associated with various neurological and autoimmune disorders. *H. pylori* are spiral shaped, Gram-negative bacteria that colonize the stomach. It is estimated that the global prevalence of *H. pylori* is about 44%; however, the prevalence in developing countries is higher than that in developed countries [171]. This significant difference can be attributed to higher hygiene standards and early diagnosis with intervention. These bacteria have been linked to gastric disorders including ulcers and gastric cancers as well as Guillain–Barré syndrome, autoimmune pancreatitis, and neurodegenerative diseases [172,173,174,175,176]. The mechanisms underlying the potential association between *H. pylori* infection and MS are not well understood. One hypothesis is that *H. pylori* may induce a pro-inflammatory response that triggers the development or exacerbation of MS. Another hypothesis is that *H. pylori* may modulate the gut microbiome, which in turn may affect the development and progression of MS.

A recent systematic and meta-analysis was conducted on 22 studies [177]. Of these, 17 studies had prevalence data comprising 2606 cases and 2200 controls (Table 2). The pooled prevalence of *H. pylori* was 44.1% in MS cases and 46.1% in controls, indicating a non-significant protective effect of *H. pylori* on MS [177]. However, the findings varied based on the diagnostic method used. Specifically, studies that utilized histopathological methods demonstrated a strong positive correlation, indicating that an active *H. pylori* infection could potentially increase the risk of developing MS [177]. Serological analysis of *H. pylori* infection is unable to distinguish between past and acute infection. Only current infections produce humoral and cellular immune responses that cross-react with host neuronal cells, ultimately contributing to neurodegeneration. Serological methods also have low accuracy and low specificity giving high false-positive rates [178]. Active *H. pylori* infection should be diagnosed via histology, the gold standard [179]. These results raise doubts about the relationship between *H. pylori* and MS and highlight the importance of conducting large-scale, well-designed prospective studies that use more precise diagnostic methods to establish any potential association.

### 4.2. C. pneumoniae

*Chlamydophila pneumoniae* is a common, obligate intracellular bacterium that can cause respiratory disease, with up to 70% of the adult population carrying antibodies [197]. *Chlamydia* species can cause persistent infections in different cells, particularly in monocytes and macrophages, leading to tissue damage through immune-mediated mechanisms such as cytokine secretion and oxidative stress. However, it is unclear if the bacteria are carried into inflammatory tissues by infected cells. *C. pneumoniae* has been associated with various chronic diseases, including asthma, diabetes, cardiovascular inflammatory disease, arthritis, and chronic obstructive pulmonary disease [198]. There is evidence to suggest the potential role of *Chlamydia pneumoniae* as a causative agent of multiple sclerosis as well as Alzheimer’s dementia, but the exact nature of this relationship remains unclear [199].

A causal role of *C. pneumoniae* involvement in MS pathogenesis is supported in part by biological (culturing), molecular, immunological, and seroepidemiological studies. The first report of an association between *C. pneumoniae* and MS came almost 20 years ago [200]. While initial studies have reported higher rates of *C. pneumoniae* detection in MS patients compared to healthy subjects or those with other neurological diseases, subsequent studies have yielded conflicting results due to variations in laboratory methods and small sample sizes. After the isolation of *C. pneumoniae* from the CSF of MS patients, Sriram appeared to show a convincing link between *C. pneumoniae* and MS [201]. Sriram et al. reported that 97% of CSF samples from MS patients were positive for *C. pneumoniae*, compared to only 18% from controls [202]. The same group also found CSF anti-*C. pneumoniae* IgG detected by ELISA in 86% of MS patients compared to 0% in patients with other neurological diseases. After these studies, other groups found evidence to suggest a causative role [203,204], while most failed to detect *C. pneumoniae* in patients with MS [205,206,207,208,209,210,211,212]. Munger and colleagues employed a nested, age-matched case–control design in their study [213]. This approach addressed certain limitations encountered in prior research, including small sample sizes, the use of nonstandardized or insensitive measures for detecting infection, lack of proper blinding, inadequate control populations, and exclusion of specific subtypes of MS. However, it is important to note that the conclusions were still limited by the fact that nearly all of the blood samples were collected after onset of the disease [213]. Consequently, the lack of prospective studies, and hence the inability to establish a temporal relationship between infection onset and MS development, leaves room for the possibility of an abnormal immune response attributed to MS itself or reactivation of latent infection [214]. Diverse studies present conflicting viewpoints on the involvement of *C. pneumoniae* in multiple sclerosis. One perspective suggests that *C. pneumoniae* merely acts as an innocent bystander within the CNS, resulting from the ongoing inflammation in MS [215]. This viewpoint proposes that the selective infiltration of infected-mononuclear cells in the CNS is favored by MS-related inflammation. On the other hand, other studies indicate that *C. pneumoniae* may function as a cofactor in both the development and progression of the disease. These studies argue that *C. pneumoniae* enhances a pre-existing autoimmune response in a specific subset of MS patients, which is supported by recent immunological and molecular evidence [205,216,217].

### 4.3. Borrelia burgdorferi

*Borrelia burgdorferi* is among the various bacterial pathogens proposed as etiologic for multiple sclerosis. Lyme borreliosis is a multisystemic tick-borne illness caused by the neurotropic spirochete, *Borrelia burgdorferi*. Symptoms of Lyme borreliosis may mimic multiple sclerosis and other CNS conditions, including polyneuropathy, encephalitis, brain tumors, and psychiatric illness. In some instances, Lyme borreliosis, sometimes referred to as the “new great imitator,” seems to be indistinguishable from MS [218]. One theory is that *Borrelia* infection may trigger an autoimmune response in some people, leading to the development of MS. This is based on the observation that both Lyme disease and MS are associated with an abnormal immune response, and that some people with MS have reported a prior history of Lyme disease or exposure to ticks. Lyme borreliosis can be misdiagnosed as MS, as demyelination is seen in both diseases.

The association between spirochetes and MS dates back to 1925 when Adams et al. conducted an experiment in which rhesus monkeys (*Macaca mulatta*) were inoculated with material from MS cases [219]. Several months later, spirochetes were observed in the ventricular fluid of these monkeys. In 1952, Steiner reported the presence of spirochetes in plaques obtained from autopsied MS patients [220]. Additionally, in 1957, Ichelson successfully cultivated spirochetes from the spinal fluid of MS patients [221]. Newman et al. replicated Ichelson’s culture method and detected spirochetes in the spinal fluid of MS patients, although in a lower percentage (18.5% compared to 78%) [222]. The recognition of Lyme arthritis in 1977 and the association between Lyme disease and chronic neurological abnormalities further strengthened the link. Detection of *Borrelia burgdorferi* DNA by PCR in the cerebrospinal fluid of patients with Lyme neuroborreliosis also contributed to this understanding [223]. Although there is evidence for a potential bacterial etiology in MS, with a particular focus on spirochetes, studies have reported conflicting results (Table 3). A meta-analysis of the association between antibiotic use and the development and progression of MS did not show a significant correlation, lending speculation to the role of bacteria in this disease [224]. Further research is thus needed to establish a more causal role between *B. burgdorferi* and MS. This appears to be a commonalty in microbe–MS associative research.

### 4.4. Mycobacterium Species

The potential link between *Mycobacterium* species and MS has garnered significant attention in the realm of MS research. *Mycobacterium* is a genus of aerobic bacteria that includes various species, some of which are known pathogens causing diseases like tuberculosis and leprosy. *Mycobacterium avium* subsp. *paratuberculosis* (MAP) emerges as another intriguing contender in the MS narrative, particularly through the lens of the molecular mimicry theory. This theory postulates that certain genetically predisposed individuals might experience an interplay wherein MAP might contribute causally to MS pathology. Currently, clinical trials involving antimycobacterial therapy targeting MAP are in progress. The first association between MAP and MS was in Sardinia, a MAP-endemic Mediterranean island [238]. A recent seroprevalence study in Japan also confirmed the association [239]. *Mycobacterium bovis* is a member of the *M. tuberculosis* complex and is responsible for causing tuberculosis primarily in cattle; however, it can occasionally lead to tuberculosis in various other mammals, including humans [240]. Bacille Calmette–Guérin (BCG), an attenuated strain derived from *M. bovis*, serves as a live vaccine against tuberculosis. Notably, clinical trials employing the BCG vaccine as an adjuvant therapy for individuals with MS have demonstrated positive outcomes. In a crossover trial, a solitary BCG vaccination showcased a reduction in disease activity as determined by MRI in a cohort of 12 RRMS patients [241]. Given that mycobacteria are intracellular pathogens, cell-mediated immunity assumes a pivotal role in defense. While the precise mechanisms underlying BCG’s impact on neuroinflammation remain somewhat enigmatic, the notion of BCG vaccination exerting a protective influence on MS progression is widely acknowledged [242]. However, further investigations are imperative to explore the potential therapeutic utility of the BCG vaccine, particularly for individuals at risk of developing MS. It is noteworthy that the relationship between mycobacteria and the initiation as well as progression of MS might potentially vary among populations, contingent upon genetic and non-genetic elements. The domain of immune modulation as a strategy against mycobacterial infections remains relatively uncharted territory. Despite its potential, this avenue has yet to be fully explored. As research advances, untangling the intricate web of interactions between mycobacteria and the immune system offers insights that could shape novel therapeutic interventions. The complexities of these relationships underscore the imperative for sustained research endeavors to decipher the role of mycobacteria in the multifaceted tapestry of MS pathogenesis.

## 5. Conclusions

Many infectious agents have been studied for their presumed association with MS, both in disease progression and pathogenesis. The infectious hypothesis of MS faces a greater paradox than the failure to produce a causative microorganism. The conflicting literature regarding infectious agents and MS highlights the considerable challenges inherent in investigating the cause of a multifactorial, heterogeneous chronic disease in which the underlying pathological process is likely initiated years, or even decades, before the initial clinical manifestations. While it is plausible to consider microbes as a potential cause of MS, establishing a definitive relationship remains far from conclusive. The role of genetic and environmental factors in the etiology of disease adds to this complexity, and thus it becomes necessary to incorporate genetic, environmental, and infectious data in the study of MS and other neurodegenerative diseases. As personalized medicine becomes more advanced, along with the capability of incorporating larger datasets, the prospects of effective treatment and increased longevity may be realized.

## Figures and Tables

**Table 2:** *H. pylori* and MS. Prevalence datasets selected from Arjmandi et al. [177].

References	Year	Diagnostic Method	Number of MS Patients	*H. pylori* Positive	Number of Controls	*H. pylori* Positive
Gavalas et al. [180]	2007	Histology	29	24	25	12
Li et al. [181]	2009	ELISA	162	67	85	36
Zarkesh et al. [182]	2009	ELISA	210	11	200	9
Ramroodi et al. [183]	2012	Western blot	78	20	123	27
Long et al. [184]	2013	Immunofluorescence	42	31	27	16
Mohebi et al. [185]	2013	ELISA	163	88	150	110
Yoshimura et al. [186]	2013	ELISA	71	15	42	14
Cook et al. [187]	2015	Histology	44	38	20	10
Gavalas et al. [188]	2015	ELISA	139	31	139	64
Malli et al. [189]	2015	ELISA	550	73	299	64
Pedrini et al. [190]	2015	ELISA	149	34	150	49
Riskind et al. [191]	2016	Latex agglutination	139	60	68	33
Efthymiou et al. [192]	2017	ELISA	386	188	420	298
Ranjbar et al. [193]	2019	ELISA	92	66	91	78
Kiani et al. [194]	2020	ELISA	154	74	39	11
Mirmosayyeb et al. [195]	2020	ELISA	127	44	177	74
Zahedi et al. [196]	2021	ELISA	71	44	145	111

**Table 3:** Conclusions from studies linking *Borrelia* and MS.

Source	Conclusions
Adams et al., 1925 [219]	Researchers inoculated rhesus monkeys with material from MS patients. Spirochetes emerged in CSF after several months.
Steiner, 1952 [220]	Reported the presence of spirochetes in plaques obtained from autopsied MS patients.
Ichelson, 1957 [221]	New culture medium allowed for growth of spirochetes from CSF of MS cases.
Newman et al., 1958 [222]	Replicated Ichelson’s culture method and detected spirochetes in spinal fluid in 18.5% of MS patients.
Schmutzhard, 1988 [225]	There is no etiologic association between *Borrelia* and the relapsing/remitting form of multiple sclerosis.
Marshall, 1988 [226]	Medical practitioners and researchers should consider using antibiotics as treatment for MS in patients who do not respond to treatment.
Coyle, 1989 [227]	Infection with *B. burgdorferi* is rare in MS and unlikely to play a significant role in MS.
Garcia-Monco et al., 1990 [228]	Researchers evaluated 55 patients with a definite diagnosis of multiple sclerosis and found Lyme disease infection in 3.
Heller et al., 1990 [229]	ELISA assay can substantiate the diagnosis of neuroborreliosis to rule it out in MS patients with positive *Borrelia* serology.
Coyle et al., 1993 [230]	Lyme serology in MS patients with no suggestive features was unlikely to indicate neurological Lyme disease.
Lana-Peixoto, 1994 [231]	A 45-year-old MS patient was infected with *Borrelia burgdorferi,* confirmed by ELISA and Western blotting. The relationship between spirochetal infection and neurological disease could not be ascertained.
Chmielewska-Badora et al., 2000 [232]	A statistically significant relationship was found between clinically confirmed diagnosis of MS and positive serologic reaction with *Borrelia* antigen.
Cheema et al., 2019 [233]	Case report of co-occurrence of MS and psychiatric features of Lyme borreliosis.
MacLean et al., 2020 [234]	No positive serological evidence of Lyme disease was identified in MS patients in Atlantic Canada.
Stricker and Johnson, 2011 [235]	Serology or CSF testing may lead to oversight of a considerable proportion of patients with neuroborreliosis, often resulting in failure to diagnose and address a condition that resembles MS.
Vatne et al., 2011 [236]	Researchers did not detect a higher frequency of Bb antibodies in serum from patients with MS compared to controls.
Forrester et al., 2015 [237]	No geographic correlation between Lyme disease and deaths due to multiple sclerosis.

## Data Availability

No new data were created or analyzed in this study. Data sharing is not applicable to this article.
